# Annual of the Universal Medical Sciences

**Published:** 1888-11

**Authors:** 


### BOOK NOTICES AND REVIEWS.

ANNUAL OF THE UNIVERSAL MEDICAL SCIENCES, issue of 1888. Edited by Dr. Charles E. Sajous and seventy associate editors. Five volumes, three dollars per volume. F. A. Davis, publisher.

We cannot better state the purpose of this *Annual* than by quoting from its preface: "The object of this *Annual of the Universal Medical Sciences* is to collate the progressive features of medical literature at large, and clinical data from countries in which no literature exists, and to present the whole once a year in a continued form, prepared by writers of known ability." This *Annual* is, therefore, a collection in permanent shape of the best medical news of each year. It is a gigantic undertaking, and will be a useful work of reference. No data is given of homœopathic literature, the learned editors evidently considering such to be beyond the pale of medical science!

